# Lubricant film formation in rough surface non-conformal conjunctions subjected to GPa pressures and high slide-to-roll ratios

**DOI:** 10.1038/s41598-020-77434-y

**Published:** 2020-12-17

**Authors:** Jonny Hansen, Marcus Björling, Roland Larsson

**Affiliations:** 1 Division of Machine Elements, Luleå University of Technology, 97187 Luleå, Sweden; 2 Transmission Development, Scania CV AB, Södertälje, Sweden

**Keywords:** Fluid dynamics, Mechanical engineering, Metals and alloys

## Abstract

A ball-on-disc machine was employed in a highly idealised setting to study the interplay between oil film formation and surface irregularities in single-sided rough elasto-hydrodynamic lubricated (EHL) conjunctions. The tests were operated under GPa pressures and high slide-to-roll ratios in a situation where the separating gap was smaller than the combined surface roughness height. Under the initial state of solid contact interference and with the operating conditions held fixed, surfaces were found to gradually conform such that a fully separating oil film of nanometre thickness eventually developed—a thin film lubrication state known as micro-EHL. Additionally, with a previously developed approach for 3D surface re-location analysis, we were able to very precisely specify the pertained nature of surface transformations, even at the asperity scale, by comparing the post-test surfaces to those in the virgin state. The surface roughness Sq was reduced by up to 17% after running-in, while the speed required for full film EHL was reduced by a remarkable 90%. Hence, full film EHL is possible even in cases where the Λ-ratio falsely suggests boundary lubrication. This discrepancy was attributed to the way surfaces are deformed inside the contact, i.e., through the establishment of micro-EHL.

## Introduction

In this research, we address the type of lubrication mechanism found in non-conformal contacts, which, under relative motion, forms a thin separating oil film of nanometre to micrometre thickness, i.e., elasto-hydrodynamic lubrication (EHL)^1–4^. EHL contacts constitute a crucial part of rotating machinery by helping to mitigate surface distress and reduce friction. Typical machine elements where EHL can be found include gears, cam followers and rolling-element bearings. The smooth surface EHL contact problem under pure rolling motion has been widely researched throughout the twentieth century and is now adequately understood by means of practical use^5,6^. However, in many technical applications, such as gears, surfaces are not ideally smooth due to economic restrictions. Additionally, combined sliding and rolling motion is an inherent part of operation, and the EHL film developed cannot in general be made thick enough to fully separate the mating surfaces. This situation is precarious since it often leads to higher friction losses and reduced service life due to fatigue-related causes^7–10^. Moreover, with the ongoing shift to electrical vehicles (EVs)^11,12^ and to achieve sustainable use of resources, the prospects for EHL contacts are being extended to even more severe lubrication regimes.

The classical regimes of lubrication are often defined by ${\Lambda } = h_{m}/Sq$, i.e., the smooth surface minimum EHL film thickness over the root-mean-square (RMS/Sq) of the combined surface roughness^2^. Typically, the most adverse regime—boundary lubrication (BL) —is considered when ${\Lambda } \le 1$, the milder mixed lubrication (ML) regime when $1 < {\Lambda } < 3$, and the mildest and typically most preferred EHL regime when ${\Lambda } \ge 3$. These regimes are not absolute, and experimental evidence often finds EHL at much lower ${\Lambda }$ than anticipated^13–20^. Many times, this desirable performance is achieved owing to a running-in sequence^21^—a settling period when asperities are allowed to conform at the micro-scale due to wear and plastic deformations. Under this process, the tops of the most prominent asperities are removed, which causes improved local hydrodynamic load carrying capacity (HLCC) such that a continuous oil film can be maintained, even if its thickness is significantly less than the ex situ RMS roughness of the surfaces^20,22^. This intermediate mode of lubrication, located between EHL and ML, is known as the micro-EHL regime^23–26^.

At present, through the advancement of a tremendous amount of research effort, of which only some are highlighted here^27–33^, the deformations in micro-EHL have been established as associated with two main characteristic features. The first is a roughness deformation (RD) component that originates from the rolling squeeze action when a roughness feature is located at the EHL contact inlet—a local EHL constriction formed at the asperity scale^34–36^. In addition, invoked by the sliding action, an independent entrainment front—a complementary effect (CE) that propagates with the mean surface speed—is formed simultaneously^37–39^. The way these components adhere depends not only on the operating conditions but also on the structure of the surface topography^40–42^; long wavelengths are more prone to elastically deform than are short wavelengths^34^, round asperity peaks will better form HLCC than will sharp ones^43^, and deep valleys that extend transversely outside of the contact area are advantageously closed^44^, although some evidence suggests that extra pockets of lubricant in some cases can be beneficial^45,46^. On the basis of the above review of the literature, most efforts have concentrated on exploring the thin film lubrication effects in non-steel conjunctions restricted from wear and permanent deformations. However, few investigations have been dedicated to exploring what implications these micro-EHL characteristics have on the topographical evolution and recovery of the fluid film in more practical EHL conjunctions.

The objective of this research was to determine what features of an engineering surface make micro-EHL possible in non-conformal steel conjunctions. A ball-on-disc machine was employed, and the contact conditions were specified to promote the characteristic micro-EHL effects mentioned previously. The machine was operated under high slide-to-roll ratios (SRRs) and GPa pressures in a highly idealised setting where the slower member of the contact pair was rough (0.34 μm) and the faster member was significantly smoother (0.021 μm) in the surface RMS. Any tribo-chemical effects^47–49^ were minimized by the use of a neat synthetic lubricant. The friction and electrical contact resistance (ECR) were simultaneously measured to monitor changes in contact performance associated with running-in. The main purpose of the running-in sequence was to produce a quasi-static state of micro-EHL from an initial state of vast topographical interference. A previously introduced very precise surface re-location routine^20^ was utilized to investigate what in the structure of the surface topography was subjected to change to form a completely separating EHL film. Here, we put forward a novel semi-analytical formula that, in addition to the already established EHL theory, requires only asperities of hemispherical shapes as input to estimate the contact performance in micro-EHL. The findings should appeal to readers that aim to understand the complex deformations ubiquitous in the film formation and collapse process of rough surface rolling/sliding EHL contacts.

## Results and discussion

The first sections provide the rolling/sliding running-in tests, while the succeeding sections place special emphasis on investigating what transformations the surfaces were required to undergo to shift from BL to full film EHL, according to the classical definitions of the lubrication regimes.

### Ball-on-disc running-in tests

**Figure 1:**
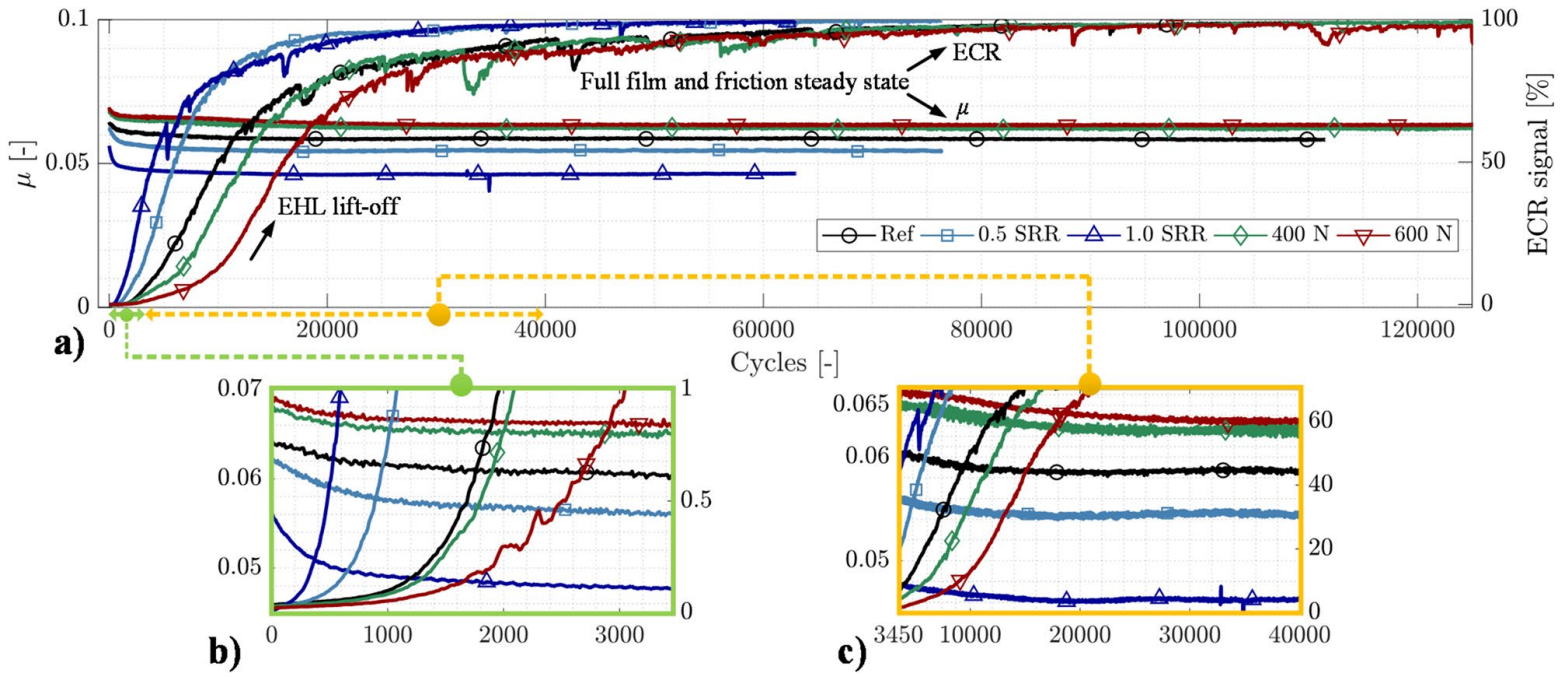
Ball-on-disc running-in test for hydrodynamic lift-off: (**a**) the entire test sequence showing friction and ECR traces, (**b**) 0–3500 ball rotating cycles—first friction reduction plateau with associated ECR lift (enlarged view) —and (**c**) 3450–40 000 ball rotating cycles—second running-in stage towards steady-state friction and ECR (micro-EHL).

Running-in results obtained from a ball-on-disc WAM (Wedeven Associates Machine) are displayed in Fig. 1. Both the coefficient of friction (*μ*) and ECR curves are shown. The ECR signal indicates the absence or degree of metallic contact. A high value close to 100% indicates full film EHL and an insignificant wear rate. A signal value below 100% indicates various degrees of fluid film rupture and thus the possibility of wear.

The operating conditions for the reference, Ref, case were 200 N (1.69 GPa maximum Hertzian pressure) and an SRR of 0.2. The entrainment speed was 1.052 m/s, and the inlet oil temperature was held fixed at 40 °C. Two additional tests were conducted to examine the influence of the SRR. In these tests, the SRR was increased to 0.5 and 1.0, while the other parameters were unchanged with respect to the Ref. Additionally, two more tests were conducted to examine the influence of an increased load from Ref, i.e., 400 N and 600 N, corresponding to maximum Hertzian pressures of 2.13 and 2.44 GPa. The Ref, 1.0 SRR, and 600 N cases are of primary concern, and the surfaces derived from those tests will later be subject to a detailed topographical analysis.

The thermally corrected^50,51^ film thicknesses calculated according to the Dowson & Hamrock^5^ equation were $h_{m} = \left\{ {0.189,{ }0.174,{ }0.184} \right\}$ and $h_{c} = \left\{ {0.330,{ }0.304,{ }0.320} \right\}$ *μ*m for the Ref, 600 N and 1.0 SRR cases; see Hansen et al.^20^ for details. The corresponding ${\Lambda }$ parameters were calculated to be ${\Lambda }_{{{\text{pre}}}} = \left\{ {0.55,{ }0.51,{ }0.54} \right\}$ using the composite surface roughness of the ball and disc specimens reported in the ‘Materials and Methods’ section of this report. Thus, BL is applicable according to conventional theory^2^. However, as seen in the friction coefficient of Fig. 1, this classification does not always apply but appears rather deceptive for the tests presented here. We therefore refer to ${\Lambda } > 0.5$ as ML from this point on.

In general, several distinct trends can be seen in both the friction and ECR behaviours in Fig. 1. The running-in process is characterized by a rapid drop in friction and a corresponding increase in ECR. In all tests, most of the running-in occurs rapidly, before 1000 cycles, although some running-in remains until approx. 40 000 cycles. The second run-in stage (compare the end of Fig. 1b with the start of Fig. 1c) has been observed and discussed previously^20^, although the mechanisms that govern the temporary asymptotic behaviour remain unclear. Additionally, a true state of running-in, i.e., where the asperity contacts are minimal, can only be accomplished at approximately 50 k cycles and above, as shown in Fig. 1a (where the ECR approaches 100%).

The mechanism involved in the overall friction reduction originates from an ongoing improvement in lubrication as surfaces gradually conform at the asperity scale^43,52,53^. Wear and plastic deformation at prominent asperities continuously improve the HLCC, and as a result, the EHL lift-off ultimately enables a shift in performance from the ML regime to the more favourable micro-EHL regime. At the end of the tests, full film separation is established due to the running-in process, and the coefficient of friction is entirely dominated by the lubricant shear stress. Note, however, that the steady-state friction is quasi-static, i.e., it still depends on the surface roughness under full film EHL^20^.

Furthermore, when the load is increased, the steady-state friction coefficient increases, while increasing the SRR has the opposite effect; see, e.g., > 40 000 ball rotating cycles (x-axis) in Fig. 1a. Given that friction is governed by the lubricant rheological properties in EHL, these observations are by no means surprising. In fact, increasing the load is expected to increase the effective viscosity (lubricant shear stress) and therefore friction^54^. Additionally, increasing the SRR is expected to cause thermal softening of the lubricant^54, 55^ and thus reduce the lubricant shear stress and friction. With this in mind, one may more easily comprehend the fact that the increased SRR component causes a larger reduction in friction with running-in, although the overall steady-state level is distinctly lower compared to both the Ref and the cases of increased load. Conversely, the case of increased load undergoes a smaller friction reduction despite the overall friction coefficient being greater than the Ref value. This situation can be more easily observed in the magnified view of Fig. 1b. The total drops in friction at the end of the test for the Ref, 600 N, and 1.0 cases were 0.064–0.058 (− 9.5%), 0.070–0.064 (− 8.7%), and 0.056–0.047 (− 17.1%), respectively. This drop in friction is believed to be closely linked to the surface transformations that enable EHL lift-off.

Interestingly, although an increase in the SRR causes a larger drop in friction, the running-in appears distinctly faster than the Ref, as seen in the earlier lift in the ECR signal for the 0.5 and 1.0 SRR cases. By contrast, increasing the load causes an increasing number of cycles before the steady state of both the friction and ECR is established. The rate of wear ($\dot{d}$) is assumed to be proportional^56^ to the normal pressure ($p$) and sliding velocity (${\Delta }u$) and inversely proportional to the hardness ($H$), i.e., $\dot{d} \propto p \times {\Delta }u/H$. Therefore, an increase in both the SRR and load should result in earlier hydrodynamic lift, i.e., given the threshold limit for how much a surface is required to transform to onset EHL. However, the effect of an increased load on the friction and ECR steady states is by no means a straightforward matter. What then remains, given that the ${\Lambda }$ ratios are fairly similar, are, e.g., the changes in micro-hardness. Although not very well explored in rough EHL contacts^57,58^, work hardening of plastically deformed asperities will affect the film formation rate under running-in, mainly by lowering the rate at which asperities flatten and thus allowing for prominent asperities to form electrical junctions for an extended period of time. Another possibility is that the Hertzian contact length is 44% wider for the 600 N case than for the 200 N case. That is, the extended running-in could simply be a result of the fact that the asperities have a longer distance to travel, thus increasing the risk of collapse anywhere within the EHL conjunction.

### Topographical transformations for EHL

The Ref, 1.0 SRR and 600 N cases are given further attention in terms of a surface analysis. From this point, the two latter cases are simply termed SRR and Load for convenience. The reference case is termed Ref as previously described. Note that any changes to the smooth disc counter surfaces were insignificant (< 5 nm) and hence will not be further discussed.

**Figure 2:**
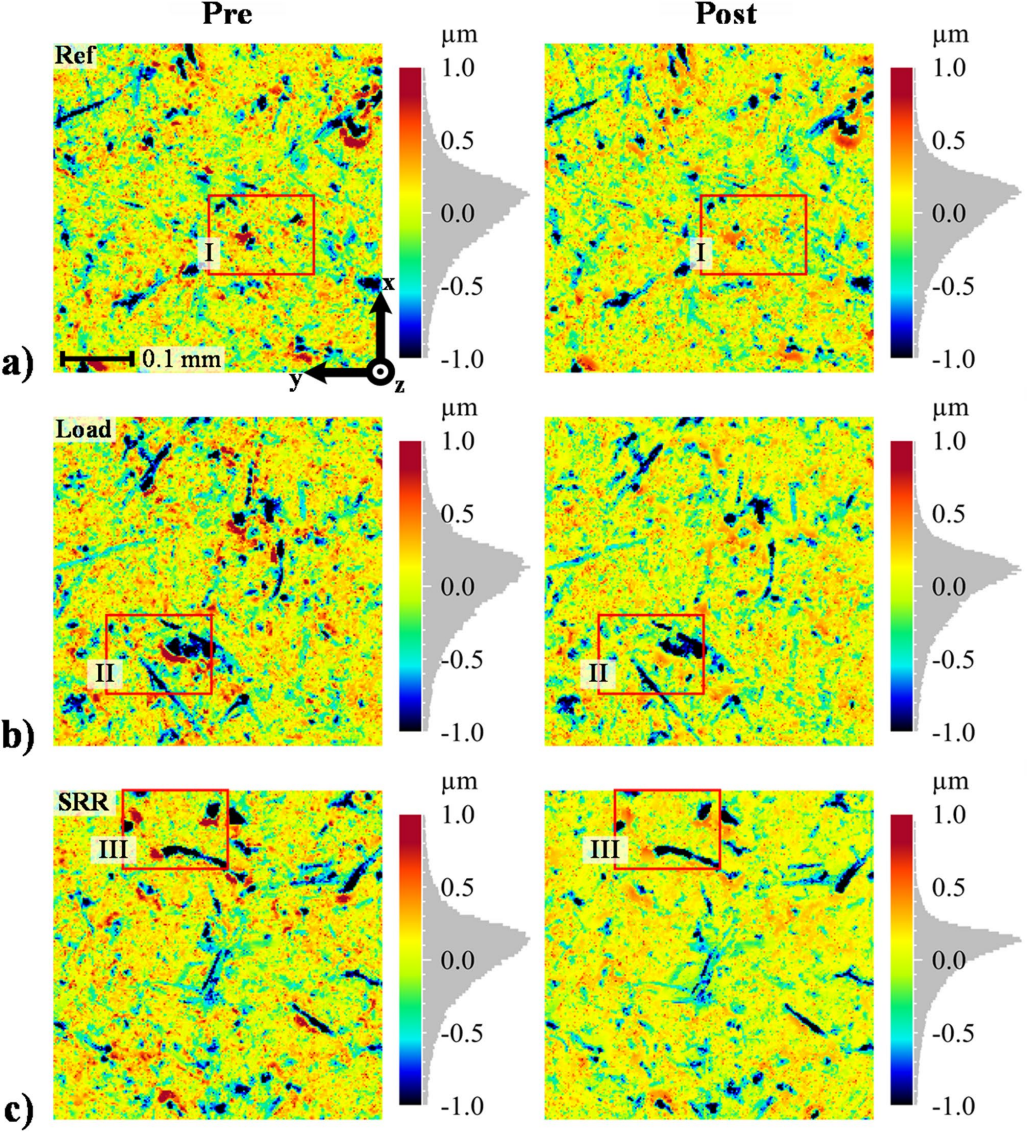
3D optical profiles of the exact same surfaces before and after the running-in tests: (**a**) the reference case (200 N, 0.2 SRR), (**b**) the case with increased load (600 N, 0.2 SRR), and (**c**) the case with increased SRR (200 N, 1.0 SRR).

Figure 2 displays the re-located pre- and post-test surfaces for the three cases. The x-direction, see Fig. 2a (pre) for the coordinate system, indicates the direction of lubricant entrainment (and sliding), whereas the y-direction represents the transverse direction. All surfaces started the running-in test in the ML regime and ended the test in the full film EHL regime. Thus, the surfaces contain information about what transformations are required to onset full film EHL from a state of vast contact interference. Examining the optical profiles, wear is clearly mild, and the main structure of the surfaces remains after the test. This applies to all cases. However, the red areas (prominent asperities) are clearly reduced as a result of running-in, although no surface stands out at first appearance. Considering the amplitude probability curves (projected on the colour bars), the upper-most tail is removed for all cases after the test, and the SRR case seems to have been subjected to the greatest changes, followed by Load and Ref. However, comparing the upper-most tail of the post-test amplitude probability curves to the computed minimum film thicknesses (which was less than 0.2 µm in all test cases), the highest asperity peaks are clearly not removed to the extent required for full film EHL. Hence, as far as conventional EHL theory is concerned, substantial contact interference is still apparent after the completed running-in tests. The ${\Lambda }$ ratios after running-in were ${\Lambda }_{{{\text{post}}}} = \left\{ {0.60, 0.57, 0.61} \right\}$, i.e., an almost indistinguishable change compared to ${\Lambda }_{{{\text{pre}}}}$ (presented previously) and thus still indicating ML. Similar trends were found and discussed in detail by the authors^20^, where it was identified that EHL is made possible only by in-contact deformations of the most prominent asperities.

### Asperity deformations

The rectangle markings labelled I, II, and III in Fig. 2 indicate 0.15 × 0.10 mm sized areas that contain particularly prominent asperities. These sites suffer from high residual stresses due to plastic deformations that under continued cyclic loading may lead to micro-pitting^9,59^. Therefore, examining these areas in further detail is of profound interest.

**Figure 3:**
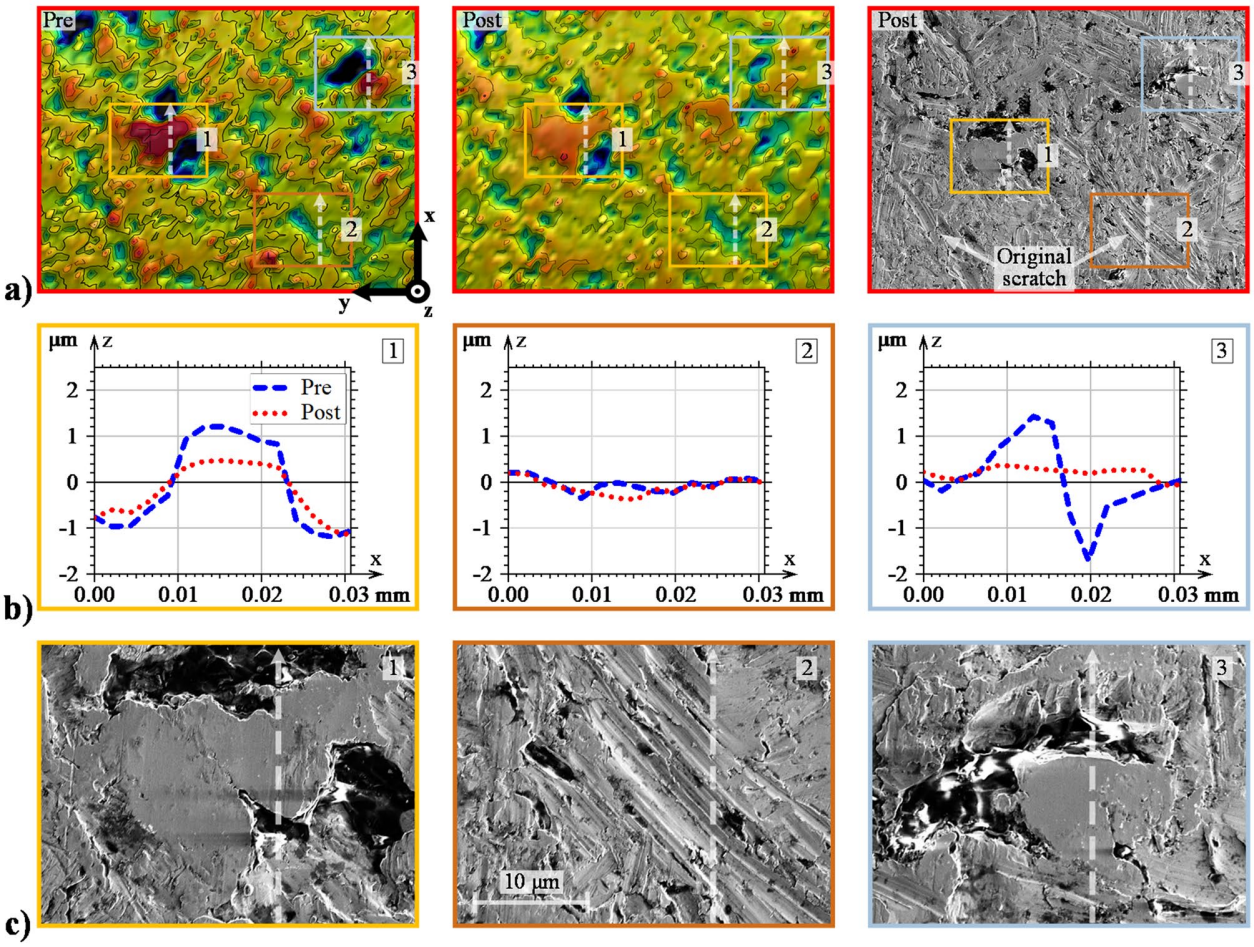
Detailed evaluation of the Ref-derived asperities: (**a**) enlarged view of area I in Fig. 2, (**b**) 2D profiles of prominent asperities taken in the vertical direction (direction of sliding), and (**c**) SEM analysis of the corresponding asperities.

**Figure 4:**
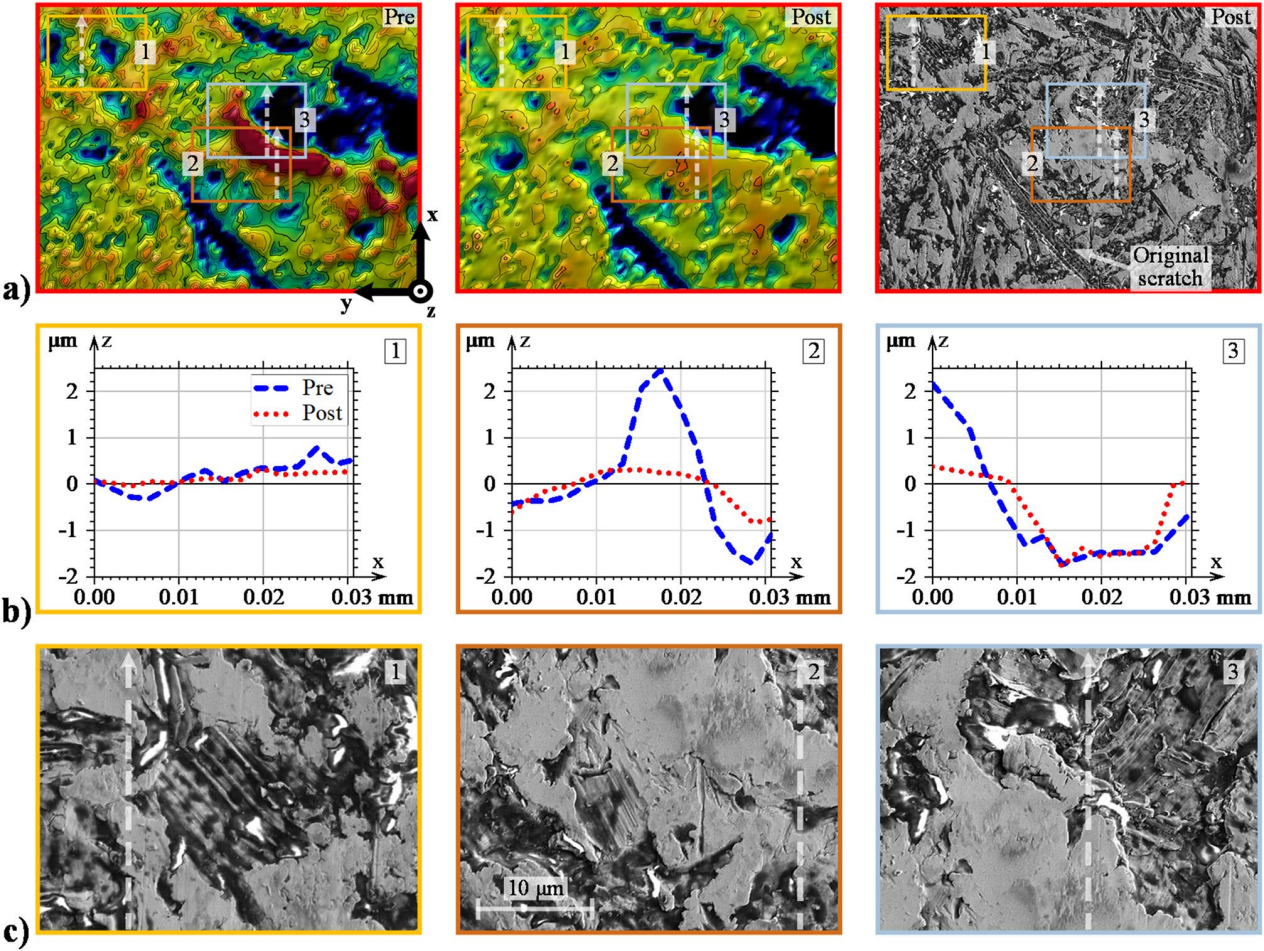
Detailed evaluation of the load-derived asperities: (**a**) enlarged view of area II in Fig. 2, (**b**) 2D profiles of prominent asperities taken in the direction of sliding, and (**c**) SEM analysis of the corresponding asperities.

**Figure 5:**
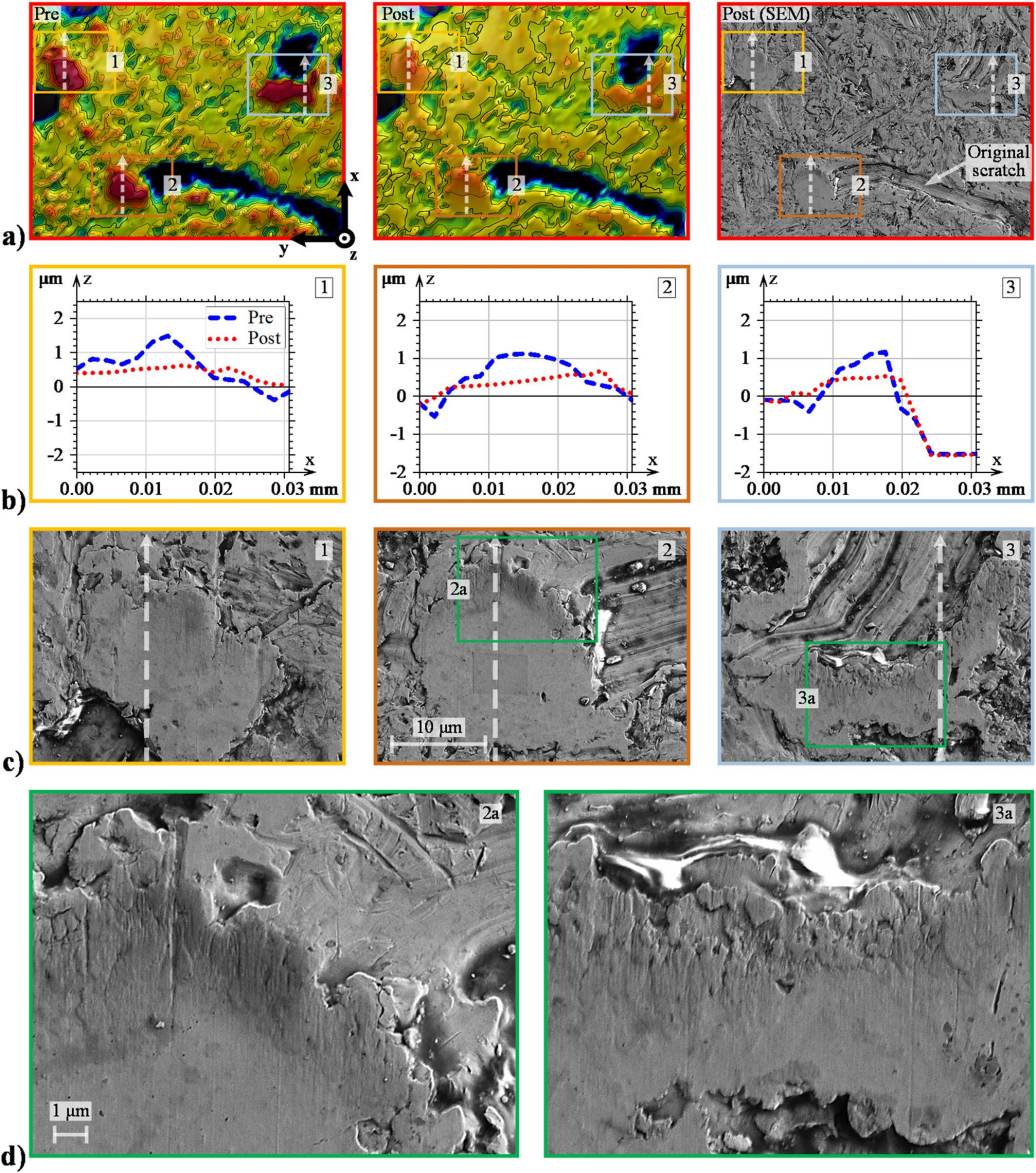
Detailed evaluation of the SRR-derived asperities: (**a**) enlarged view of area III in Fig. 2, (**b**) 2D profiles of prominent asperities taken in the direction of sliding, and (**c**) SEM analysis of the corresponding asperities.

Figure 3a displays an enlarged view of area I in Fig. 2a i.e., pre- and post-test analysis of the Ref case. The post-test area was also examined by SEM (see the right-most upper figure). The SEM analysis was included to bring forth an additional level of detail about the surface degradation modes that optical profilometry may fail to discern. Three squared areas, labelled 1, 2, and 3, are highlighted for even closer examination. Figure 3b shows roughness profiles taken in the direction of sliding and lubricant entrainment (x-direction). Figure 3c shows the corresponding areas under high magnification SEM analysis. Similar figures were also compiled for the 600 N case (Fig. 4) and the 1.0 SRR case (Fig. 5) to reveal how the normal and tangential forces affect wear and plastic flow at the local asperity scale.

As described in the previous section, asperities undergo plastic deformations under the initial rotations when the breakdown of the EHL film is most severe. This most likely occurs within the initial 1000 cycles; recall Fig. 1b. Similar trends have been observed elsewhere^20,43,60^. Presumably, what then follows is mild wear that polishes the upper-most asperity tops until EHL lift-off occurs. Examination of the most prominent asperities shows clear evidence of this occurrence; see, e.g., area 1 in Fig. 3, area 2 in Fig. 4, and all three areas in Fig. 5. In general, prominent asperities have been plastically deformed by the normal load component such that material has flowed uniformly in both the direction of the rolling/sliding and in the transverse direction. The no. 1 asperity in Fig. 3b is a prime example of how material has flowed in both the leading and trailing directions of rolling. Similar observations can be made from the profiles highlighted in Fig. 4b and Fig. 5b. Hence, 3D plastic flow does seem to be a dominant feature for any asperity above a certain datum, aptly, the minimum film thickness, which was approximately 0.2 μm in all three cases. Equivalently, an initial ${\Lambda }$ of approximately 0.5 clearly yields plastic deformations and very mild wear, mainly to the most prominent asperities, regardless of the load and SRR. Reasonably, increasing the load should facilitate material flow in both lateral directions, while increasing the SRR should promote material flow in the direction of sliding. This scenario does indeed seem to be the case, although arriving at such conclusions may be difficult when individual asperities are evaluated, especially since asperity deformations depend on their initial shapes, as well as their surrounding areas. Therefore, at a later stage of this report, we evaluate the directionality of roughness from a more quantitative-oriented perspective.

Furthermore, note that under SEM, the Ref post-test asperities appear almost completely smoothened, and any obvious signs of abrasive wear are absent. Similar observations can be made in the 600 N case, albeit with the asperities appearing even more flattened. When the SRR was increased to 1.0, directionality in the asperity deformations became more apparent. In particular, the examined asperities appear to have deformed into micro-wedges, with a converging gap formed in the direction of entrainment. This observation is perhaps better viewed in the profiles of Fig. 5b. Whether wedge formations are a general manifestation of the imposed sliding action is unclear. However, these implications are indeed interesting since a converging gap should reasonably lead to an improved HLCC. Additionally, a less desirable outcome of directionally skewed asperities is the increased risk of micro-pitting, which is known to propagate in the direction opposite that of sliding^8^. Another notable feature of the SRR-derived surface is that the flattened asperities appear to have developed small (micro-scale) pits at the leading edges; see Fig. 5d, which displays a further magnified view of the 2nd and 3rd asperities. The reason for this damage is unclear. Potentially, it could be a result of early-stage micro-pitting, or it could be a result of surface degradation due to cavitation^61^.

Furthermore, in both optical profiles, as well as in the SEM micro-graphs of, e.g., part **a** in all the above figures, the original scratches from the machining process still remain after all three test cases. Generally, the surface degradation is so mild that low-amplitude features are only scarcely modified; see, e.g., the no. 2 profiles in Fig. 3b and the no. 1 profiles in Fig. 4b, which both show low-amplitude roughness that has been levelled out to some extent. Valley features, on the other hand, appear virtually unaffected as long as they are deep and wide enough; see, e.g., the no. 3 profiles in both Figs. 4 and 5b. Note, however, that unaffected valleys are not a general outcome of the running-in process. The exception arises when material flows from adjacent asperities. In that case, the valleys may be completely filled in. The no. 3 asperity profile in Fig. 3b shows an example of this occurrence. At first appearance, the material seems to have flowed in the rolling/sliding direction to cover the adjacent valley located at x = 0.2. However, as may be realized by considering the corresponding area in Fig. 3a, the material has more likely flowed from a larger feature located to the far right (in the horizontal direction).

### 3D surface roughness parameters

Having evaluated the deformations of individual asperities, we now consider the transformations of the surface topographies previously considered from a typical surface-metrology-oriented approach. This approach should bring forward more easily distinguishable characteristics imposed by the normal load and the increased SRR component.

A 3D surface roughness analysis using MountainsMap was conducted to determine the evolution of roughness due to running-in. The computations were made over the same areas before and after the test according to Fig. 2. Several commonly reported parameters for the Ref-, Load- and SRR-derived surfaces are shown in Table 1. Note that a more extensive list of surface roughness parameters is provided in Supplementary Tables S3–S8 and that the corresponding average parameters for Table 1 are given in Supplementary Tables S1 and S2 (nine measurements conducted around the run-track before and after testing). Similar to our previous study involving similar ball surfaces^20^, the evolution of roughness of the initial and re-located areas is fairly representative of the overall run-track.

**Table 1:** Surface roughness parameters evaluated on exactly the same area before and after running-in.

Case	Sq^a^ (μm)	Ssk^a^ (–)	Sku^a^ (–)	Sp^a^ (μm)	Sv^a^ (μm)	Sal^b^ (μm)	Sdq^c^ (°)	Ssc^d^ (1/mm)
**Ref**								
Pre	0.349	− 1.01	7.16	1.86	2.79	9.08	8.81	112
Post	0.312	− 1.48	7.46	1.15	2.48	9.19	8.15	98.9
**Load**								
Pre	0.351	− 0.78	6.59	2.63	2.18	9.64	8.88	114
Post	0.302	− 1.55	7.36	1.05	2.23	11.19	7.71	94.0
**SRR**								
Pre	0.353	− 1.47	9.22	1.68	3.13	9.12	8.58	106
Post	0.300	− 2.24	12.17	0.97	3.55	10.12	7.32	79.4

^a^Height: Root-mean-square average (RMS/Sq), skewness (Ssk), kurtosis (Sku), maximum peak height (Sp), maximum pit height (Sv).

^b^Spatial: Autocorrelation length (Sal).

^c^Hybrid: Root-mean-square gradient (Sdq).

^d^Feature: Arithmetic mean summit curvature (Ssc).

Considering the data presented in Table 1, a clear trend imposed by the different operating conditions can be observed. The SRR-derived topography underwent the greatest changes in Sq (15%), followed closely by Load (14%) and Ref (11%). The change in the Sq parameter is particularly interesting since it is typically used for estimating the contact quality, ${\Lambda } = h_{m}/Sq$. For this reason, we note that for all the evaluated pre-test topographies, the EHL/ML transition was measured to occur at speeds above 12 m/s. This speed was the maximum one considered prior to the start of the running-in tests (see Materials and Methods section for details). Nevertheless, in all cases, full film EHL occurred at 1 m/s after the running-in tests. The EHL/ML transition was thus reduced by more than 12 times in terms of the entrainment speed. The speed relates to the Hamrock-Dowson minimum film thickness as $h_{m} \propto u_{e}^{0.68}$; thus, this speed reduction may cause a film thickness reduction by more than 80%. The fact that the Sq has only been reduced by approximately 10–15% is thus quite remarkable and indicates a strong mismatch in the value of ${\Lambda }$ which forms the full film regime boundary. The missing factor for proper representation of the lubricating quality is most likely the consideration of how asperities deform due to the hydrodynamic pressure. We highlighted the improved micro-conformity, such as slopes and curvatures, as a key factor of the onset of the micro-EHL regime in our previous investigations^18,20^. Similar findings are presented here; the Sdq {15, 13, 8%} and the Ssc {25, 18, 12%} parameters follow the same rankings as those of the Sq. Hence, considering that the reduction in the Sdq and Ssc parameters is either similar to or greater than that of the Sq, the changes in these parameters must play a vital part in the feasibility of full film EHL at 1 m/s.

Generally, a lower slope indicates a larger area for an asperity to carry a lubricant film. That is, when asperities flatten, valleys are closed, and larger patches of areas are formed that can develop hydrodynamic pressure^62^. As a result of the improved hydrodynamic pressure build-up, elastic amplitude reduction^34^ at these sites may be enhanced, and asperities become able to pass the conjunction without rupture of the lubricant film, although conventional theory (${\Lambda }$) still suggests vast contact interference.

### Slopes and radii

Thus far, the analysis has shown that the shift in the lubricating regime from ML to EHL was feasible due to deformations of the most prominent asperities. Much of the data, such as valleys, passed the running-in tests essentially unchanged. Thus, to enhance the topographical changes imposed by the increased sliding action and the normal load, the quantitative analysis will proceed with consideration of the highest asperity peaks, which was achieved by thresholding the material-ratio curve at the 65% highest peaks of the pre-test surfaces. The absolute height level was then used to threshold the corresponding post-test surfaces for appropriate comparison.

**Figure 6:**
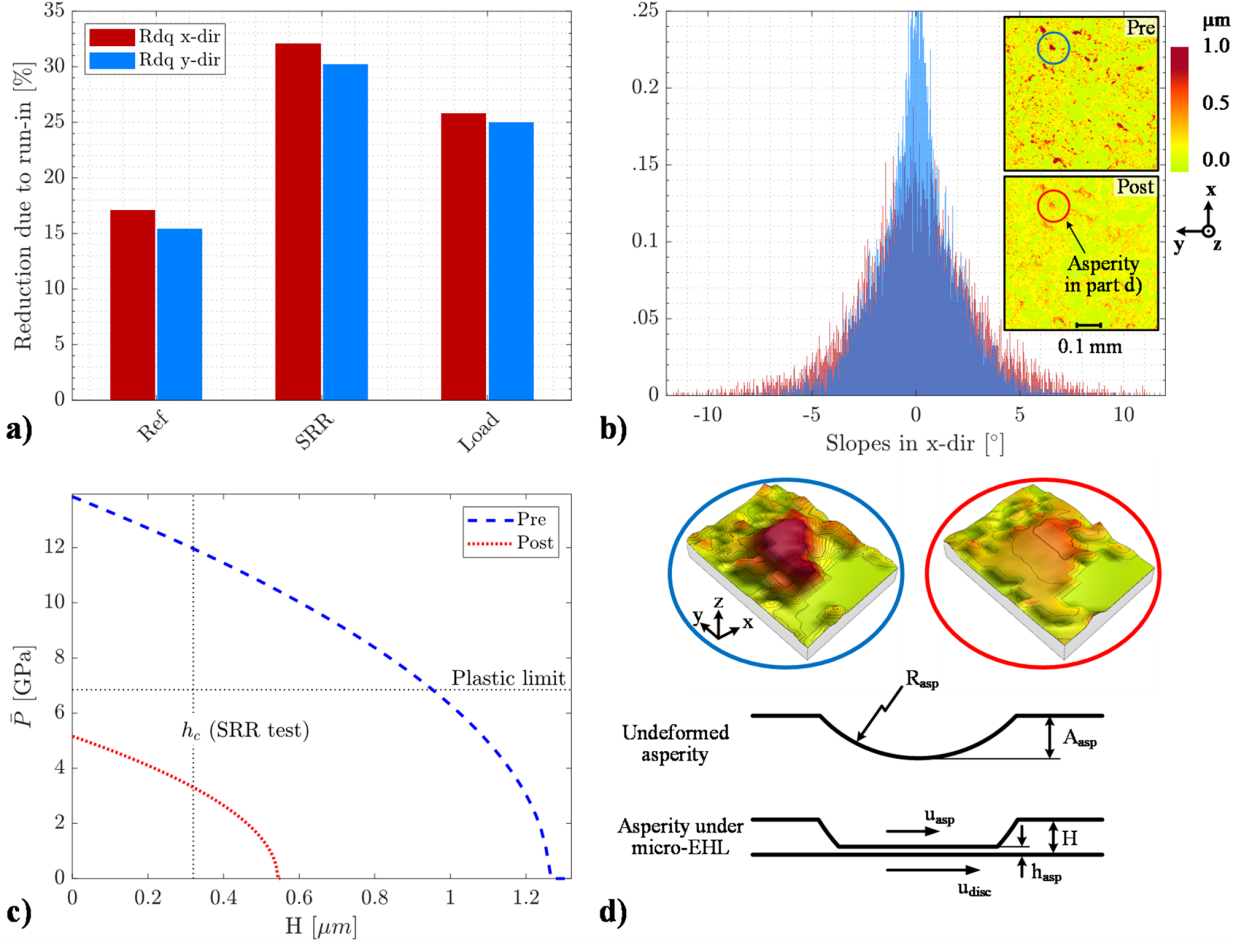
**a**) The Rdq distribution in both the x- (sliding) and y-directions, (**b**) the surface pair after extraction of the 65% highest peaks together with the histogram of slopes in the x-direction, (**c**) the solution to the proposed micro-EHL model, i.e., the mean hydrodynamic pressure acting on the idealised version of the pre- and post-test asperity shown in (**d**), and additionally in (**d)**, schematic including the essential features of the micro-EHL model.

Of particular interest is to determine whether a general outcome of rolling/sliding running-in is that the normal load component promotes plastic flow in both lateral directions and whether the SRR component mainly promotes plastic flow in the direction of sliding. One way to bring about these details is to examine the RMS slope, Rdq, of the surfaces separately in both the x- and y-directions. Figure 6a displays the percent reduction in the Rdq in both directions due to running-in for all three test cases. In contrast to the Sdq parameter, which was calculated using MountainsMap, the Rdq was calculated using a central difference gradient method in MATLAB. As before, the computations were made over the surfaces shown in Fig. 2, but now (as stated previously) solely over the highest peaks to enhance the effect of running-in. The specific pre-test amplitudes were then used as a threshold for the corresponding post-test surface to facilitate an appropriate comparison.

From Fig. 6a, the rankings clearly follow that of the Sdq parameter presented in Table 1, i.e., the greatest changes in asperity slopes occur for the SRR case, followed by the Load and Ref cases. Interestingly, in all cases, the slopes in the x-direction have undergone the greatest changes, thus indicating directionality in the direction of rolling and sliding. This result is also confirmed by the Str parameter, which indicates less isotropy after the test (see Supplementary Tables S3–S8). Presumably, this outcome indicates that the slopes in the entrainment direction are more important for EHL than those in the transverse direction. This result is expected when consulting amplitude reduction theory. Note, however, that similar observations were recently made for close to pure rolling EHL contacts^52^. Thus, to what extent the directionality is driven by the sliding action is unclear. However, it is clear that the SRR case causes the greatest difference between the Rdq in the x- and y-directions, thus confirming that the SRR component skews asperities more than the other test cases, presumably due to the higher tangential forces. By contrast, the smaller difference for the 600 N case suggests more uniform plastic deformations of asperities in both lateral directions. Hence, with increasing normal load, the pressing effect from the normal load clearly predominates running-in.

For the above reasons, one should investigate whether any predominance of positive or negative slopes may be distinguished. A predominance of positive slopes (diverging heights) would indicate the formation of beneficial wedges, as was discussed with regards to Fig. 5. Figure 6b shows the normalized PDF (probability density function) histogram of slopes in the x-direction comprising the SRR surfaces. Note that the analysed (thresholded) surface pair of Fig. 2c is shown in the inset plot and that the corresponding coordinate system and colour bar are shown to the right. As illustrated, the change in the distribution is essentially uniform, and any difference in the positive and negative slopes is difficult to distinguish. Note, however, that by close visual inspection of the SRR-derived surface, when the pre- and post-images in Fig. 2c are alternated in view, material appears to pile up in the direction of sliding at several of the prominent asperities, as shown in the profiles of Fig. 5b. These effects may generally be obscured by the randomness of isotropic surfaces and may be more easily distinguishable under, e.g., a surface of distinct transverse roughness lay, such as those found in gear contacts. Nonetheless, the entire distribution of the slopes clearly needed to be reduced for micro-EHL to be established. The reduction is predominated by plastic deformations, and wear mainly affects the asperity peaks.

### A simple model for micro-EHL

On the basis of the above analysis, asperities with high slopes and amplitudes or, equivalently, small tip radii have undergone the greatest changes for the establishment of full film EHL. Generally, these sharp asperities are at risk of film rupture and of suffering detrimental contact pressures that may cause loss of service life due to contact fatigue. Hence, if the relation between these geometrical entities and film formation can be understood, it would be possible to design surfaces for optimal lubrication quality. Actually, given that the default model to estimate lubricating quality still requires consideration of the ${\Lambda }$ parameter, a vast need exists for a simplified but much more accurate approach.

In fact, the contact behaviour due to the asperity slopes, radii, heights and local oil film thickness can be evaluated in a simplified manner by combining the idealised micro-EHL model proposed by Fein and Kreuz^23^ with some more recent advances in EHL research. The micro-EHL model is depicted in the schematic of Fig. 6d. The inset plot corresponds to the asperity labelled no. 2 in Fig. 5a. In principle, if the separation from the basal plane, $H$, is less than the height of an asperity, then changes in the micro-EHL film thickness, $h_{asp}$, must alter the asperity deformation. Then, from Hertzian elastic theory, one can show^5^ that the mean pressure at an asperity with initial undeformed amplitude $A_{asp}$ and with radius $R_{asp}$ can be computed from

1 $$\overline{P} = \frac{2}{3\pi }E^{\prime}\left( {\frac{{A_{asp} }}{{R_{asp} }}} \right)^{1/2} \left( {1 + \frac{{h_{asp} }}{{A_{asp} }} - \frac{H}{{A_{asp} }}} \right)^{1/2}$$

where $E^{\prime}$ is the effective elastic modulus (231 GPa). Conveniently, Choo et al.^35,61^, in an extension of the work by Guangteng et al.^63^, found that a micro-EHL film thickness developed under an asperity is closely related to the macro-contact central film thickness, $h_{c}$, according to the following ratio:

2 $$\frac{{h_{asp} }}{{h_{c} }} = \left[ {\frac{{u_{asp} }}{{u_{ball} }}} \right]^{0.67} \left[ {\frac{{R_{asp} }}{{R_{ball} }}} \right]^{0.464}$$

where $u_{asp}$ and $u_{ball}$ are the tangential speed of the asperity and the ball, respectively, and $R_{ball}$ is the ball radius. Similar postulates were put forward by Jacobson^22^. Moreover, when the asperity is attached to the ball surface, the first term is unity, and the film thickness ratio depends only on the radii. Then, if $h_{asp}$ in Eq. (2) is substituted into Eq. (1), and if $H$ is taken as $h_{c}$, then the following semi-analytical expression can be deduced:

3 $$\overline{P} = \frac{2}{3\pi }E^{\prime}\left( {\frac{{A_{asp} }}{{R_{asp} }}} \right)^{1/2} \left[ {1 + \frac{{h_{c} }}{{A_{asp} }} \times \left( {\left( {\frac{{R_{asp} }}{{R_{ball} }}} \right)^{0.464} - 1} \right)} \right]^{1/2}$$

Now, by inserting the pertinent asperity radii and heights, one can use Eq. (3) to approximate the mean pressure acting on an asperity attached to the ball surface due to a micro-EHL film when the asperity is located in the central part of the EHL conjunction. The radii before and after the test were approximated by fitting a circular arc to the no. 2 profiles in Fig. 5 (neglecting any wedges), with the resulting values being 0.015 and 0.045 mm, respectively. The corresponding heights were approximately 1.2 and 0.5 µm, as shown in the same figure. Since the model requires only the geometry of a hemispherical asperity, in addition to the well-established EHL theory, it could serve as a useful rule of thumb for assessing the elastic response due to different combinations of asperity heights and radii. Strictly speaking, Eq. (3) is flawed since, e.g., both non-Newtonian effects at the asperity tips and fluid entrapment due to inlet squeeze need to be accounted for to capture precise details of the elastic deformation. Despite this, the proposed model may be useful since it reveals the main features associated with micro-EHL oil film formation.

Figure 6c shows the solution to Eq. (3) as a function of the smooth central EHL film thickness. The central film thickness variation corresponds to the following entrainment speed interval of 1.05–12 m/s. The black dashed and dotted lines mark the central film thickness in the running-in tests and the limit for the occurrence of plastic deformation, respectively. Indeed, with consideration of the pressure responses of the pre- and post-test asperities, both the asperity heights and their slopes can be regarded as governing geometrical entities that determine whether the deformations are detrimental in nature or if the much more favourable micro-EHL state is attained. The blue dashed line shows the pressure response for the pre-test asperity. As shown, when the central film thickness is thin enough (~ 1.2 µm) to cause interference of the undeformed asperity, the elastic deformation undertakes the development of the micro-EHL film. As a result, the asperity pressure continuously increases with the narrowing of the macro-EHL film. Evidently, the pre-test asperity suffers pressures high enough to cause plastic deformations of very thick films—of course, this is invalid since a plastic model is lacking. At the running-in operating film thickness (0.32 µm), the contact interference is vast, and the contact pressure is well within the plastic domain. Notably, for the post-test asperity (red dotted line), the contact conditions are greatly mitigated due to running-in, and the deformations are entirely elastic in nature. In terms of slopes, this result is quite remarkable considering that the asperity shown in Fig. 6d had to reduce only slightly, to approximately 0.95° after the test from an initial 2.3°. Hence, if the slopes of these asperities could be reduced within a few degrees in the machining process, then plastic deformations resulting from operation could be avoided. This outcome would presumably have far reaching effects on operation since plastic deformations may cause contact fatigue and loss of service life, e.g., by micro-pitting^59^.

### Spectral distributions

Unsurprisingly, the asperity slopes and summit radii are interconnected to the harmonics of the surface waviness—a change in slopes (or radii) must therefore also cause changes to the surface spectral content. The surface spectral content is well known to affect EHL film formation^26^. Long wavelengths will be more susceptible to elastic deformations, whereas short wavelengths will pass the conjunction essentially undeformed. Therefore, one must evaluate in what way the spectral content of the different test cases is required to change to enable micro-EHL after running-in.

**Figure 7:**
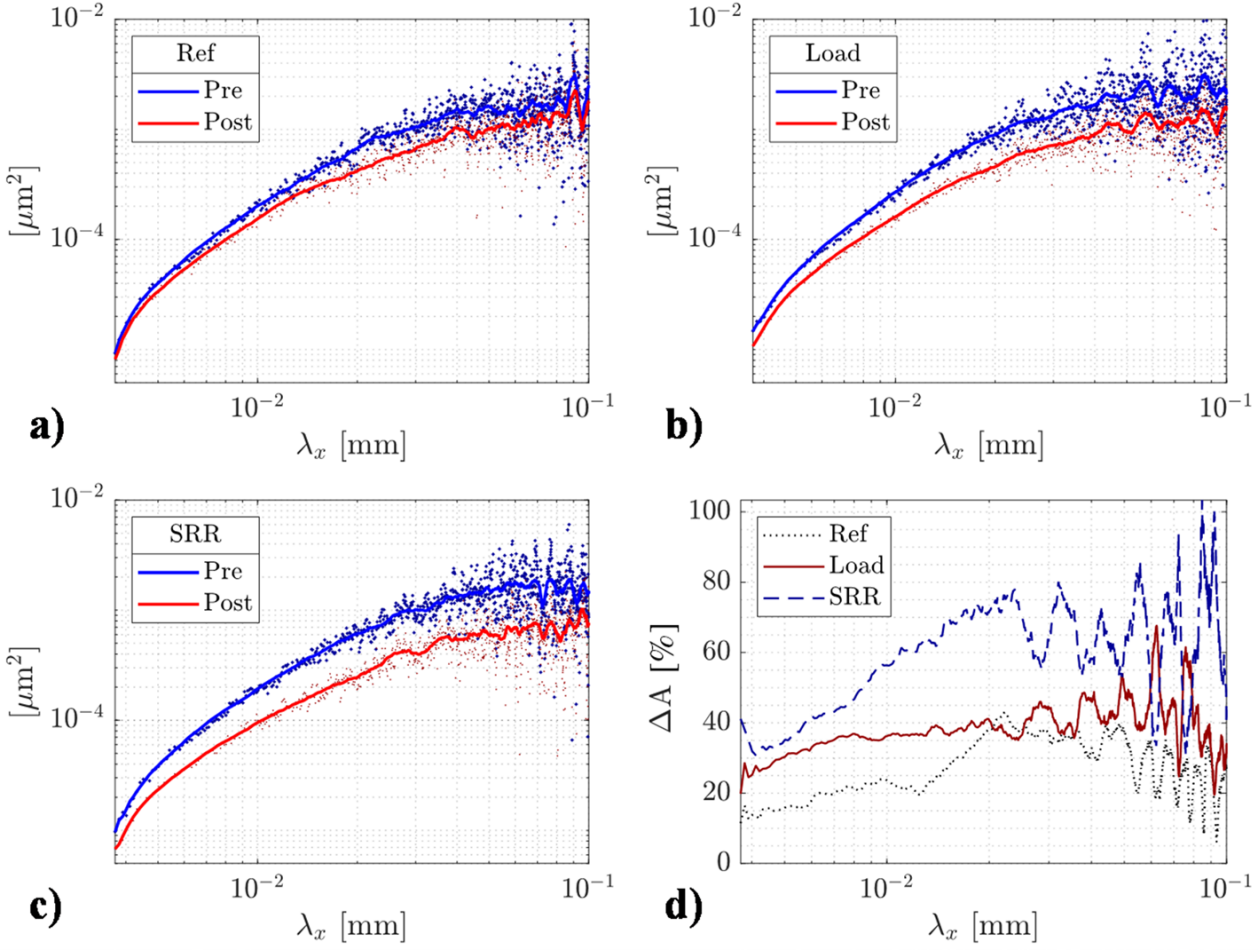
PSD (amplitude versus spatial wavelength) curves showing the spectral distribution of the 65% highest peaks of the pre-test and re-located surfaces. The FFT analysis was conducted in the direction of entrainment (and sliding) to detect any residues from the film formation process: (**a**) Ref, (**b**) Load, (**c**) SRR, and (**d**) all compared by percent difference.

The spectral distributions before and after the test computed in the x-direction (entrainment) are shown in Fig. 7a (Ref), b (Load) and c (SRR). The spectral distributions were obtained using the PSD (power spectrum density) feature in MountainsMap. The cloud of dots represents the entire data set, while the curves represent the corresponding smoothed data (moving average with a span of 50 data points). Note that the analysis was conducted on an extended length scale (0.8 mm in the x-direction) to ensure robustness of the PSD for spatial wavelengths up to 0.1 mm. Considering the Ref case first, the entire spectrum of wavelengths is clearly more or less affected—compare the red curve (post-test) to the blue curve (pre-test). The reduction in amplitudes less than 0.1 mm confirms that small-scale waviness is removed as a result of running-in. Notably, changes to wavelengths even less than 0.01 mm can be detected using the present surface re-location approach. Presumably, these represent the flattening of asperities observed in SEM micro-graphs; see section ‘Asperity deformations’. With an increased normal load, a similar, although more enhanced, reduction in amplitudes can be observed over the entire spectrum of wavelengths. The effect appears even more enhanced with increased SRR. The changes in spectral content can be more easily observed in Fig. 7d, which shows the percent difference in amplitude, where ${\Delta }$A is a function of the wavelength in all three cases. From this plot, an increased normal load clearly affects all wavelengths more than in the Ref, and increasing the SRR yields even greater changes to all wavelengths.

The decay in ${\Delta }$A with increasing wavelength (> ~ 0.05 mm) —compare, e.g., the Ref case with the Load curve in Fig. 7d—is quite interesting and may possibly, at least in part, be attributed to the amplitude reduction of the spectral components (RD). For clarity, let us consider the amplitude reduction effect as proposed by Venner and Lubrecht^34^. We do not repeat the formula here since it has been presented and evaluated on a similar rough surface in detail elsewhere^20^. However, for the reference operating conditions, it can be shown that amplitudes that belong to wavelengths of approximately 0.01, 0.1 and, e.g., 1 mm will be reduced to approximately 95, 70 and 10% of their original values. Increasing the load is expected to increase the deformations further still, although quite marginally—to 95, 60, and 5%, respectively. Although the longer wavelength components are more prone to amplitude reduction and thus can potentially avoid wear, they may at the same time be less prone to plastically deform in EHL. This is the case since roughness features having longer wavelengths (equivalent to larger tip radii or less slope if the amplitude is fixed) develop less hydrodynamic pressure, $\overline{P}$, according to Eq. (3). Hence, given that the ${\Delta }$A in Fig. 7d shows some tendency towards zero for wavelengths approaching 0.1 mm, the deformations may be shifting from a predominance in wear and/or being mainly plastic (either due to direct metallic contact and/or excessive hydrodynamic pressure in EHL) towards becoming more elastic. The effect is by no means unrealistic, but a highly sophisticated fully deterministic EHL simulation would be required to prove the presence, significance, and evolution of these effects under the conditions considered here. We will leave this for a future study.

For the SRR case, a more complex situation supposedly arises in the way the film interacts with the surface roughness. Experimental evidence appears to suggest that an increase in the SRR will not distinctly affect the amplitude reduction of the roughness features itself^35,36,61^ but rather the CE that propagates with the mean entrainment speed^37^. In particular, the CE wave will be stretched since the roughness features are located for a longer time within the contact inlet. The wavelength extension can be estimated accordingly; see $\lambda_{CE} = \lambda \times u_{e} /u_{ball}$, where $\lambda$ is the initial harmonic wavelength and $u_{e}$ is the entrainment speed. Inserting the pertinent speeds, we find that the roughness that corresponds to 1.0 SRR will be stretched considerably ($u_{e} /u_{ball} = 2.0$) compared to the Ref and increased load cases (1.1). Therefore, film breakdown and subsequent wear should affect longer wavelengths more in the SRR-derived surface compared to the Ref and 600 N cases. Considering the changes in the SRR-derived spectrum, see Fig. 7d, which appears to be the case indeed. At the shortest wavelength considered (3.7 μm), the amplitudes have reduced to a comparable extent relative to the 600 N case. However, with increasing wavelength, the ${\Delta }$A curve increases significantly more than that of the Load, and the effect becomes even more pronounced at the longest wavelengths (0.1 mm). The finding is novel and perhaps the first quantitative evidence, derived from experimental work, that the complementary wave indeed may be an active mechanism in the lubrication of engineering-rough rolling/sliding EHL contacts and that it may cause permanent changes to surface topographies after running-in.

As a closing remark, we emphasize that Fig. 7 suggests that the removal of the small-scale roughness, i.e., the shortest wavelengths imposed on the roughness profile (< 0.1 mm), is responsible for the greatly improved lubrication quality achieved after running-in. This idea is especially manifested in the fact that the ${\Lambda }$ parameter, which is the conventional way of estimating the state of lubrication, increased only marginally. Recall that before the test, ${\Lambda }_{{{\text{pre}}}} = \left\{ {0.55,{ }0.51,{ }0.54} \right\}$ for {Ref, SRR, Load}, whereas after the test, ${\Lambda }_{{{\text{post}}}} = \left\{ {0.60, 0.57, 0.61} \right\}$. In fact, in a preliminary study^64^ based on the numerical work in^65^, we found that under similar operating conditions, the overall minimum film thickness, $h_{mm} = {\text{min}}\left[ {min\left( h \right)} \right]$– not to be confused with the side lobe minimum film thickness—has a strong negative linear correlation to the small-scale surface roughness (the shortest wavelengths). When small-scale roughness (small enough to maintain Sq ≈ 0) is incorporated into the smooth ball, $h_{mm}$ will reduce much more than the Sq of the incorporated small-scale roughness. If the overall (total) Sq is increased to 0.5 µm and the Sq of small-scale roughness is further increased, but with maintained global Sq, we find that $h_{mm}$ will continue to reduce in a linear manner until, presumably, film breakdown occurs. Thus, the way spectral content is modified as a result of running-in, in combination with the asperity radii, has emerged from the present investigation as crucial for the establishment of micro-EHL and, consequently, for the materialization of EHL friction.

## Conclusions

This paper concerns the interaction between surface roughness and the formation of micro-EHL from the perspective of the running-in of non-conformal contacts. A high-precision ball-on-disc machine, lubricated with neat synthetic base oil, was operated under GPa pressures and under different rolling and sliding combinations to manifest characteristic differences in the way the oil film develops and maintain a quasi-static steady state of lubrication. 3D surface re-location analysis was employed to determine how surfaces and individual asperities must transform to onset micro-EHL from a state of vast contact interference. The main conclusions are summarized accordingly.

- Based on ECR measurements, EHL lift-off occurs faster when the SRR is increased and is delayed when the load is increased. The latter is unexpected if one assumes wear to follow an Archard type of law and that a threshold limit exists for how much a surface is required to transform to onset full film EHL.
- The surface slope and curvature parameters were identified as either equal to or more sensitive than the RMS surface height, i.e., the Sq parameter. Thus, the RMS height and slope were put forward as key parameters for the establishment of full film lubrication in the micro-EHL regime, which was found to occur at ${\Lambda } = 0.6$. A lowering of the slope with maintained height indicates a larger area for an asperity to carry a lubricant film and therefore improves the local HLCC.
- A semi-analytical expression that describes the pressure response acting on an asperity in micro-EHL was introduced. Employed on one of the asperities in the present investigation, the deformations were shown to be initially plastic in nature but become fully elastic at the completion of running-in. The expression is easy attainable since it requires only an asperity of hemispherical shape as input in addition to the already established EHL theory.
- A microscopic examination of the surface topographies revealed that the plastic deformations are essentially omni-directional in nature. However, directionality in the rolling sliding direction was found when the roughness data were analysed separately in the transverse direction and the direction of rolling and sliding. The uni-directionality was enhanced when the slide-to-roll ratio was increased, whereas the omni-directionality was enhanced from the pressing action when the load was increased.
- The entire spectrum of wavelengths was essentially affected for micro-EHL establishment. Small-scale waviness, i.e., “micro-roughness”, was particularly reduced and was therefore highlighted as pertinent for adequate lubrication. A hypothesis was proposed stating that the amplitude reduction of waviness affects the spectral distribution. When the slide-to-roll ratio was increased, long wavelengths were more affected than when the load was increased. This effect could originate from the stretching of wavelengths when the roughness is located in the EHL contact inlet, i.e., due to the CE imposed by the sliding action.

With controlled slopes and small-scale roughness, the study at hand reveals that plastic deformations of surface irregularities can be minimized as the micro-EHL film becomes maximized. For technical applications such as the gears found in transmission assemblies, the economic prospects of these outcomes may be far reaching, especially as fuel economy and durability become increasingly important in the ever-increasing pursuit of sustainable resource use.

## Materials and methods

**Figure 8:**
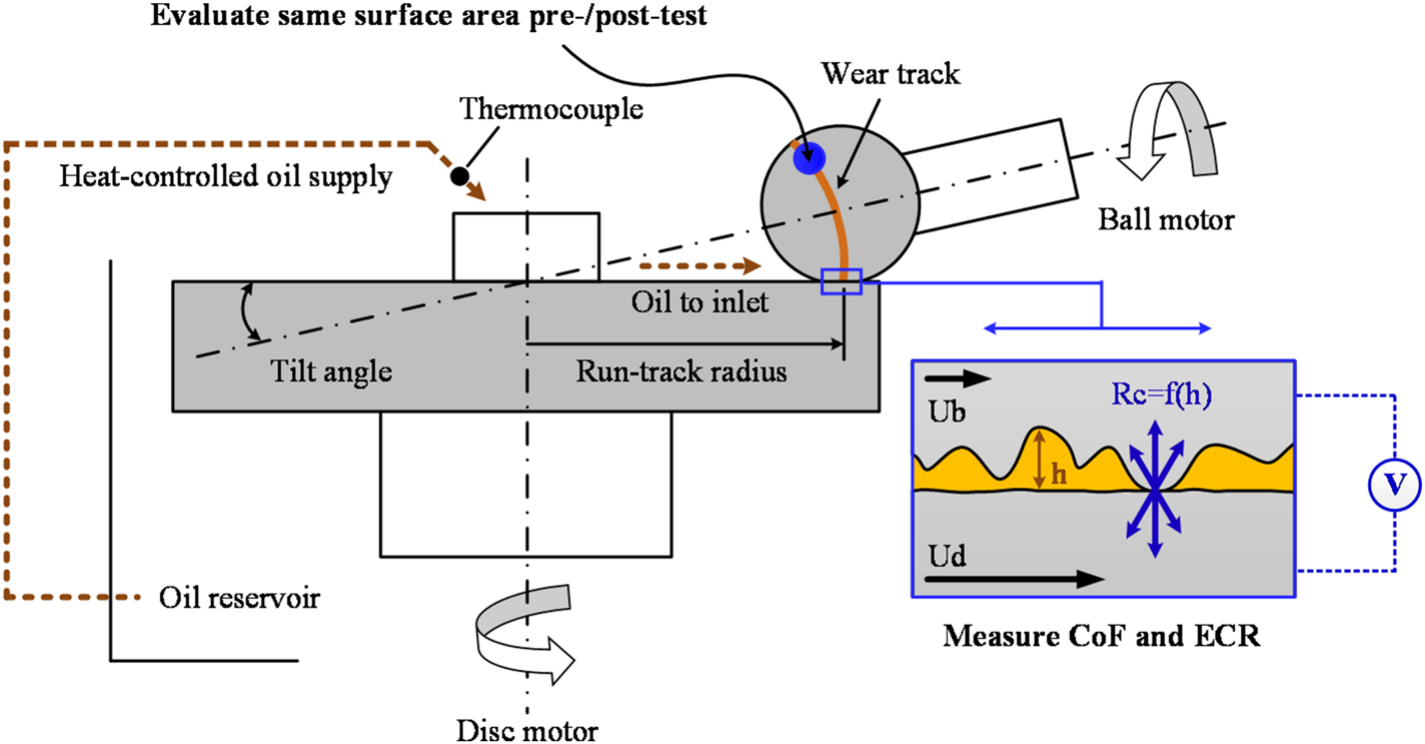
The most essential components of the experimental setup and procedure (not to scale).

A WAM ball-on-disc machine equipped for measurement of the coefficient of friction (CoF or *μ*) and ECR was employed in this investigation; see Fig. 8 for a schematic. The test specimens, running-in procedure, and procedure for the acquisition of surface topographies will only be briefly summarized here to avoid repetitive descriptions. The overall procedure is similar to that published previously; see Hansen et al.^20^, which provides a more extensive outline.

### Test specimens

The ball and disc specimens were prepared by Wedeven Associates Inc., Edgemont Pa, USA. They were made of through hardened DIN 100Cr6 (AISI 52100) ball bearing steel with approximately 60 HRC (6.85 GPa). The elastic modulus and Poisson’s ratio were 210 GPa and 0.3, respectively. The ball and disc surface topographies were prepared with an isotropic surface finish. The ball specimens were prepared from 13/16 in (20.64 mm) diameter, grade 10, standard ball bearings. The disc was polished smooth to an average Sq (RMS) of 0.021 ± 0.005 μm, while the balls were considerably rougher, i.e., an average Sq of 0.34 ± 0.014 μm.

The lubricant was a specifically prepared non-commercial poly-alpha-olefin (PAO) from Agrol Lubricants, Sweden. The lubricant was a neat base oil and thus contained no tribo-improving additives, which confined film formation effects solely to the interplay between the lubricant entrainment and the surface topographies.

### Test protocol and procedure

Three main test cases were considered: one reference case (termed Ref), one case with an increased load (termed Load) and one case with an increased slide-to-roll ratio (termed SRR); see Table 2. The operating conditions for the Ref case were 200 N (maximum Hertzian pressure of 1.69 GPa), a SRR of 0.2, an entrainment speed of 1.052 m/s (mean tangential surface speed), and a fixed steady-state inlet oil temperature of 40 °C. In the Load case, the normal load was increased to 600 N (2.44 GPa), and in the SRR case, the SRR was increased to 1.0. Note that in all cases, the rough ball moved slower than the smooth disc, i.e., $SRR = 2 \times \left( {u_{d} - u_{b} } \right)/\left( {u_{d} + u_{b} } \right)$, where *u* refers to the tangential speeds of the ball (*b*) and disc (*d*).

**Table 2:** Main tests under examination. Note that two more tests were conducted to capture the progressive influence of the load (400 N, 0.2 SRR) and SRR (200 N, 0.5 SRR) on running-in.

Case	Abbreviation	Load (N)	SRR (–)	Temperature (°C)	Entrainment speed (m/s)
I	Ref	200	0.2	40 °C	1.052
II	SRR	200	1.0	Same	Same
III	Load	600	0.2	Same	Same

The test rig components were thoroughly cleaned with heptane and rinsed with ethyl alcohol before each test. Fresh PAO was then added to the oil reservoir, and the lubricant was heated to thermal stability for a duration of approximately 1 h under continuous circulation of the oil through the closed-loop system. Oil was continuously supplied to the oil dispenser at a rate of 200 mL/min during the entire test sequences to ensure fully flooded conditions. A new run-track radius and a new ball surface were chosen so that each test was conducted with a new contact pair. The entrainment speed was ramped up to 12 m/s where the desired load was applied. The machine was subsequently calibrated to minimize contact spin before the desired SRR was applied. Using an automatic routine that was previously developed by the authors^20^, the speed was then gradually reduced to a fixed running-in speed. In this way, any pre-test running-in effects were minimized.

The test sequence was initiated at the specified entrainment speed, and the operating conditions were held constant until the completion of running-in. This part of the tests is shown in the present investigation. Both the friction coefficient and the ECR signal were monitored during the entire test sequences. Running-in was considered completed when the ECR signal had reached close to 100%. A 100% ECR value indicates that an insignificant number of electrical passages, i.e., an insignificant number of asperity collisions due to fluid film collapse, were formed during the evaluated time interval (1 kHz data samples averaged over 1 s). Thus, at this occurrence, the conjunction was considered completely separated by a fully developed EHL oil film. Electrical breakdowns were not expected since PAO is not electrically conductive^18^. An ECR value below 100% indicates occasional contact within the evaluated time interval and thus operation within either the BL or ML regimes.

### Surface re-location and metrology

A 1.40 by 1.05 mm area of the ball surface (rough surface) was measured before each test using a Zygo NewView 7300 3D optical surface profilometer (Zygo Corporation). The measurements were conducted with 10 × magnification and a 0.5 × field of view, providing a resolution of 640 × 480 pixels of the unprocessed data. The same area was subsequently re-located after the test to evaluate surface roughness changes due to running-in. Additionally, surfaces were also evaluated at 9 randomly distributed areas around the wear track before and after each test to determine the representability of the re-location analysis to the entire ball run-track. The disc specimen was measured at 8 evenly distributed areas around the circumference and at 3 radial positions (3 × 8 positions). The specimens were cleaned with heptane and rinsed with ethyl alcohol prior to all topographic measurements.

The acquired data were processed using MATLAB R2019b (MathWorks) and MountainsMap Premium 7.4 (Digital Surf). The evaluation of the post-test surfaces was confined solely to the run-track, which was achieved by masking out data that were located outside of the Hertzian contact dimension. The ball pre- and post-test surface roughness data were corrected for both rotation and translation misalignments, as well as for misalignments in altitude. The ball curvature was removed using a 2^nd^ degree polynomial, and all surface data were filtered using a robust Gaussian filter with a 0.8 mm cut-off (nesting index). Scales larger than the specified cut-off were not considered relevant for the formation of the EHL oil film (hence filtration), while the smallest scale was limited by the sampling interval, 2.19 μm, i.e., the smallest spatial component of uncertainty, or resolution. The resolution is sufficient to resolve significant peaks and even smaller-scale roughness irregularities. Hence, a single-scale analysis was considered sufficient to indicate the relative changes in the surface roughness parameters with running-in and to resolve those prominent roughness features that were mainly involved in the formation of the micro-EHL oil film. Note, however, that the ability to fully understand the relationship between surface roughness parameters and the phenomena that influence their evolution in use may require measurement and characterization at multiple scales^66^. These actions may be appropriate in a future study.

Regarding the SEM analysis, a Zeiss Sigma VP (Carl Zeiss Microscopy GmbH, Germany) field emission scanning electron microscope (FE-SEM) in secondary (SE2) electron mode was employed for further evaluation of the individual asperities. The accelerating voltage was set to 3.0 kV to retrieve information mostly from the upper-most surface area. The specimens were ultrasonically cleaned in acetone and rinsed with ethyl alcohol prior to evaluation in a high vacuum chamber. The asperities were evaluated under magnifications down to 20 k.

## Supplementary information

Supplementary Information.
