# Supplementary material for: Lubricant film formation in rough surface non-conformal conjunctions subjected to GPa pressures and high slide-to-roll ratios
Source: Sci Rep. 2020 Dec 17;10:22250. doi: 10.1038/s41598-020-77434-y (PMC7746704; doi:10.1038/s41598-020-77434-y)
Supplement: Supplementary file 1 — Supplementary Information. [file 41598_2020_77434_MOESM1_ESM.docx]

Supplementary Information for

**Lubricant film formation in rough surface non-conformal conjunctions subjected to GPa pressures and high slide-to-roll ratios**

**Jonny Hansen^1,2*^, Marcus Björling^1^ & Roland Larsson^1^**

^1^ Division of Machine Elements, Luleå University of Technology, Luleå, SE-97187, Sweden.
^2^ Transmission Development, Scania CV AB, Södertälje, Sweden.
***Correspondence and requests for materials should be addressed to J.H. (email:** [**jonny.hansen@scania.**](mailto:jonny.hansen@scania.)**com)**

# Averaged surface roughness analysis (9 measurements per ball)

**Supplementary Table 1.** Average surface roughness parameters evaluated on nine positions around the ball run-track **before** running-in. Standard deviations in parenthesis.

| Case | Sq^a)^  [$\boldsymbol{\mu}$m] | Ssk^a)^  [-] | Sku^a)^  [-] | Sp^a)^  [$\boldsymbol{\mu}$m] | Sv^a)^  [$\boldsymbol{\mu}$m] | Sal^b)^  [$\boldsymbol{\mu}$m] | Sdq^c)^  [$\boldsymbol{^{\circ}}$] | Ssc^d)^  [$\boldsymbol{1/}$mm] |
| --- | --- | --- | --- | --- | --- | --- | --- | --- |
| Ref | 0.330  (0.017) | -0.866  (0.154) | 6.56  (0.65) | 2.01  (0.30) | 2.77  (0.31) | 8.81  (0.57) | 8.90  (0.31) | 119  (4.9) |
| Load | 0.335  (0.013) | -0.945  (0.253) | 7.30  (1.11) | 2.28  (0.58) | 2.80  (0.35) | 9.34  (1.18) | 8.88  (0.15) | 118  (4.8) |
| SRR | 0.342  (0.010) | -0.835  (0.143) | 7.25  (1.24) | 2.62  (0.52) | 2.95  (0.42) | 9.83  (0.40) | 8.89  (0.12) | 119  (2.8) |
| *^a)^Height: Root-mean-square average (RMS/Sq), skewness (Ssk), kurtosis (Sku), maximum peak height (Sp), maximum pit height (Sv). ^b)^Spatial: Autocorrelation length (Sal). ^c)^Hybrid: Root-mean-square gradient (Sdq). ^d)^Feature: Arithmetic mean summit curvature (Ssc).* | | | | | | | | |

**Supplementary Table 2.** Average surface roughness parameters evaluated on nine positions around the ball run-track **after** running-in. Standard deviations in parenthesis.

| Case | Sq^a)^  [$\boldsymbol{\mu}$m] | Ssk^a)^  [-] | Sku^a)^  [-] | Sp^a)^  [$\boldsymbol{\mu}$m] | Sv^a)^  [$\boldsymbol{\mu}$m] | Sal^b)^  [$\boldsymbol{\mu}$m] | Sdq^c)^  [$\boldsymbol{^{\circ}}$] | Ssc^d)^  [$\boldsymbol{1/}$mm] |
| --- | --- | --- | --- | --- | --- | --- | --- | --- |
| Ref | 0.313  (0.014) | -1.45  (0.315) | 8.37  (3.08) | 1.17  (0.13) | 2.92  (0.44) | 9.69  (0.70) | 8.39  (0.18) | 104  (2.5) |
| Load | 0.285  (0.016) | -1.86  (0.322) | 10.3  (2.50) | 0.988  (0.074) | 2.96  (0.37) | 10.60  (0.70) | 7.34  (0.15) | 87.0  (3.6) |
| SRR | 0.302  (0.013) | -1.52  (0.179) | 8.86  (1.82) | 1.17  (0.24) | 3.33  (0.80) | 10.34  (0.71) | 7.96  (0.17) | 98.4  (2.7) |
| *^a)^Height: Root-mean-square average (RMS/Sq), skewness (Ssk), kurtosis (Sku), maximum peak height (Sp), maximum pit height (Sv). ^b)^Spatial: Autocorrelation length (Sal). ^c)^Hybrid: Root-mean-square gradient (Sdq). ^d)^Feature: Arithmetic mean summit curvature (Ssc).* | | | | | | | | |

# Surface roughness re-location analysis (1 measurement pre/post test per ball)

**Supplementary Table 3.** Surface roughness parameters of the **pre-test Ref** specimen.

ISO 25178

Height Parameters

Sq 0.349 µm Root-mean-square height

Ssk -1.01 Skewness

Sku 7.16 Kurtosis

Sp 1.86 µm Maximum peak height

Sv 2.79 µm Maximum pit height

Sz 4.65 µm Maximum height

Sa 0.251 µm Arithmetic mean height

Functional Parameters

Smr 0.687 % c = 1 µm under the highest peak Areal material ratio

Smc 0.346 µm p = 10% Inverse areal material ratio

Sxp 0.897 µm p = 50%, q = 97.5% Extreme peak height

Spatial Parameters

Sal 0.00908 mm s = 0.2 Autocorrelation length

Str 0.767 s = 0.2 Texture-aspect ratio

Std 25.0 ° Reference angle = 0° Texture direction

Hybrid Parameters

Sdq 0.155 Root-mean-square gradient

Sdr 1.18 % Developed interfacial area ratio

Functional Parameters (Volume)

Vm 1.83e-05 mm³/mm² p = 10% Material volume

Vv 0.000365 mm³/mm² p = 10% Void volume

Vmp 1.83e-05 mm³/mm² p = 10% Peak material volume

Vmc 0.000256 mm³/mm² p = 10%, q = 80% Core material volume

Vvc 0.000305 mm³/mm² p = 10%, q = 80% Core void volume

Vvv 5.96e-05 mm³/mm² p = 80% Pit void volume

Feature Parameters

Spd 3471 1/mm² pruning = 5% Density of peaks

Spc 162 1/mm pruning = 5% Arithmetic mean peak curvature

S10z 3.84 µm pruning = 5% Ten point height

S5p 1.71 µm pruning = 5% Five point peak height

S5v 2.13 µm pruning = 5% Five point pit height

Sda 0.000258 mm² pruning = 5% Mean dale area

Sha 0.000281 mm² pruning = 5% Mean hill area

Sdv 1.08e-08 mm³ pruning = 5% Mean dale volume

Shv 9.29e-09 mm³ pruning = 5% Mean hill volume

Functional Parameters (Stratified surfaces)

Sk 0.633 µm Core roughness depth

Spk 0.380 µm Reduced summit height

Svk 0.597 µm Reduced valley depth

Smr1 8.90 % Upper bearing area

Smr2 83.3 % Lower bearing area

Spq 0.268 Plateau root-mean-square roughness

Svq 0.855 Valley root-mean-square roughness

Smq 91.3 Material ratio at plateau-to-valley transition

EUR 15178N

Hybrid Parameters

Sdq 0.155 Root-mean-square slope

Sds 13277 1/mm² density of summits

Ssc 112 1/mm Arithmetic mean summit curvature

Sdr 1.19 % Developed interfacial area

Sfd 2.62 Fractal dimension of the surface

**Supplementary Table 4.** Surface roughness parameters of the **post-test Ref** specimen.

ISO 25178

Height Parameters

Sq 0.312 µm Root-mean-square height

Ssk -1.48 Skewness

Sku 7.46 Kurtosis

Sp 1.15 µm Maximum peak height

Sv 2.48 µm Maximum pit height

Sz 3.63 µm Maximum height

Sa 0.229 µm Arithmetic mean height

Functional Parameters

Smr 34.1 % c = 1 µm under the highest peak Areal material ratio

Smc 0.307 µm p = 10% Inverse areal material ratio

Sxp 0.841 µm p = 50%, q = 97.5% Extreme peak height

Spatial Parameters

Sal 0.00919 mm s = 0.2 Autocorrelation length

Str 0.763 s = 0.2 Texture-aspect ratio

Std 163 ° Reference angle = 0° Texture direction

Hybrid Parameters

Sdq 0.143 Root-mean-square gradient

Sdr 1.01 % Developed interfacial area ratio

Functional Parameters (Volume)

Vm 1.06e-05 mm³/mm² p = 10% Material volume

Vv 0.000318 mm³/mm² p = 10% Void volume

Vmp 1.06e-05 mm³/mm² p = 10% Peak material volume

Vmc 0.000243 mm³/mm² p = 10%, q = 80% Core material volume

Vvc 0.000261 mm³/mm² p = 10%, q = 80% Core void volume

Vvv 5.63e-05 mm³/mm² p = 80% Pit void volume

Feature Parameters

Spd 3832 1/mm² pruning = 5% Density of peaks

Spc 151 1/mm pruning = 5% Arithmetic mean peak curvature

S10z 2.70 µm pruning = 5% Ten point height

S5p 0.844 µm pruning = 5% Five point peak height

S5v 1.86 µm pruning = 5% Five point pit height

Sda 0.000225 mm² pruning = 5% Mean dale area

Sha 0.000252 mm² pruning = 5% Mean hill area

Sdv 9.15e-09 mm³ pruning = 5% Mean dale volume

Shv 5.72e-09 mm³ pruning = 5% Mean hill volume

Functional Parameters (Stratified surfaces)

Sk 0.562 µm Core roughness depth

Spk 0.218 µm Reduced summit height

Svk 0.559 µm Reduced valley depth

Smr1 7.03 % Upper bearing area

Smr2 81.3 % Lower bearing area

Spq 0.242 Plateau root-mean-square roughness

Svq 0.806 Valley root-mean-square roughness

Smq 91.3 Material ratio at plateau-to-valley transition

EUR 15178N

Hybrid Parameters

Sdq 0.143 Root-mean-square slope

Sds 13090 1/mm² density of summits

Ssc 98.9 1/mm Arithmetic mean summit curvature

Sdr 1.02 % Developed interfacial area

Sfd 2.67 Fractal dimension of the surface

**Supplementary Table 5.** Surface roughness parameters of the **pre-test SRR** specimen.

ISO 25178

Height Parameters

Sq 0.353 µm Root-mean-square height

Ssk -1.47 Skewness

Sku 9.22 Kurtosis

Sp 1.68 µm Maximum peak height

Sv 3.13 µm Maximum pit height

Sz 4.81 µm Maximum height

Sa 0.248 µm Arithmetic mean height

Functional Parameters

Smr 1.38 % c = 1 µm under the highest peak Areal material ratio

Smc 0.331 µm p = 10% Inverse areal material ratio

Sxp 0.945 µm p = 50%, q = 97.5% Extreme peak height

Spatial Parameters

Sal 0.00912 mm s = 0.2 Autocorrelation length

Str 0.644 s = 0.2 Texture-aspect ratio

Std 65.5 ° Reference angle = 0° Texture direction

Hybrid Parameters

Sdq 0.151 Root-mean-square gradient

Sdr 1.11 % Developed interfacial area ratio

Functional Parameters (Volume)

Vm 1.72e-05 mm³/mm² p = 10% Material volume

Vv 0.000348 mm³/mm² p = 10% Void volume

Vmp 1.72e-05 mm³/mm² p = 10% Peak material volume

Vmc 0.000251 mm³/mm² p = 10%, q = 80% Core material volume

Vvc 0.000285 mm³/mm² p = 10%, q = 80% Core void volume

Vvv 6.38e-05 mm³/mm² p = 80% Pit void volume

Feature Parameters

Spd 3266 1/mm² pruning = 5% Density of peaks

Spc 160 1/mm pruning = 5% Arithmetic mean peak curvature

S10z 4.08 µm pruning = 5% Ten point height

S5p 1.55 µm pruning = 5% Five point peak height

S5v 2.53 µm pruning = 5% Five point pit height

Sda 0.000284 mm² pruning = 5% Mean dale area

Sha 0.000299 mm² pruning = 5% Mean hill area

Sdv 1.21e-08 mm³ pruning = 5% Mean dale volume

Shv 9.85e-09 mm³ pruning = 5% Mean hill volume

Functional Parameters (Stratified surfaces)

Sk 0.585 µm Core roughness depth

Spk 0.357 µm Reduced summit height

Svk 0.639 µm Reduced valley depth

Smr1 8.67 % Upper bearing area

Smr2 81.8 % Lower bearing area

Spq 0.251 Plateau root-mean-square roughness

Svq 0.842 Valley root-mean-square roughness

Smq 88.8 Material ratio at plateau-to-valley transition

EUR 15178N

Hybrid Parameters

Sdq 0.151 Root-mean-square slope

Sds 13286 1/mm² density of summits

Ssc 106 1/mm Arithmetic mean summit curvature

Sdr 1.13 % Developed interfacial area

Sfd 2.63 Fractal dimension of the surface

**Supplementary Table 6.** Surface roughness parameters of the **post-test SRR** specimen.

ISO 25178

Height Parameters

Sq 0.300 µm Root-mean-square height

Ssk -2.24 Skewness

Sku 12.2 Kurtosis

Sp 0.970 µm Maximum peak height

Sv 3.55 µm Maximum pit height

Sz 4.52 µm Maximum height

Sa 0.209 µm Arithmetic mean height

Functional Parameters

Smr 68.0 % c = 1 µm under the highest peak Areal material ratio

Smc 0.252 µm p = 10% Inverse areal material ratio

Sxp 0.885 µm p = 50%, q = 97.5% Extreme peak height

Spatial Parameters

Sal 0.0101 mm s = 0.2 Autocorrelation length

Str 0.609 s = 0.2 Texture-aspect ratio

Std 65.3 ° Reference angle = 0° Texture direction

Hybrid Parameters

Sdq 0.129 Root-mean-square gradient

Sdr 0.810 % Developed interfacial area ratio

Functional Parameters (Volume)

Vm 8.91e-06 mm³/mm² p = 10% Material volume

Vv 0.000261 mm³/mm² p = 10% Void volume

Vmp 8.91e-06 mm³/mm² p = 10% Peak material volume

Vmc 0.000209 mm³/mm² p = 10%, q = 80% Core material volume

Vvc 0.0002 mm³/mm² p = 10%, q = 80% Core void volume

Vvv 6.13e-05 mm³/mm² p = 80% Pit void volume

Feature Parameters

Spd 2133 1/mm² pruning = 5% Density of peaks

Spc 149 1/mm pruning = 5% Arithmetic mean peak curvature

S10z 2.96 µm pruning = 5% Ten point height

S5p 0.798 µm pruning = 5% Five point peak height

S5v 2.17 µm pruning = 5% Five point pit height

Sda 0.000282 mm² pruning = 5% Mean dale area

Sha 0.000432 mm² pruning = 5% Mean hill area

Sdv 1.12e-08 mm³ pruning = 5% Mean dale volume

Shv 6.8e-09 mm³ pruning = 5% Mean hill volume

Functional Parameters (Stratified surfaces)

Sk 0.388 µm Core roughness depth

Spk 0.195 µm Reduced summit height

Svk 0.600 µm Reduced valley depth

Smr1 7.13 % Upper bearing area

Smr2 76.2 % Lower bearing area

Spq 0.201 Plateau root-mean-square roughness

Svq 0.854 Valley root-mean-square roughness

Smq 89.9 Material ratio at plateau-to-valley transition

EUR 15178N

Hybrid Parameters

Sdq 0.129 Root-mean-square slope

Sds 13023 1/mm² density of summits

Ssc 79.4 1/mm Arithmetic mean summit curvature

Sdr 0.819 % Developed interfacial area

Sfd 2.64 Fractal dimension of the surface

**Supplementary Table 7.** Surface roughness parameters of the **pre-test Load** specimen.

ISO 25178

Height Parameters

Sq 0.351 µm Root-mean-square height

Ssk -0.783 Skewness

Sku 6.59 Kurtosis

Sp 2.63 µm Maximum peak height

Sv 2.18 µm Maximum pit height

Sz 4.81 µm Maximum height

Sa 0.255 µm Arithmetic mean height

Functional Parameters

Smr 0.0667 % c = 1 µm under the highest peak Areal material ratio

Smc 0.350 µm p = 10% Inverse areal material ratio

Sxp 0.902 µm p = 50%, q = 97.5% Extreme peak height

Spatial Parameters

Sal 0.00964 mm s = 0.2 Autocorrelation length

Str 0.776 s = 0.2 Texture-aspect ratio

Std 114 ° Reference angle = 0° Texture direction

Hybrid Parameters

Sdq 0.156 Root-mean-square gradient

Sdr 1.19 % Developed interfacial area ratio

Functional Parameters (Volume)

Vm 1.8e-05 mm³/mm² p = 10% Material volume

Vv 0.000368 mm³/mm² p = 10% Void volume

Vmp 1.8e-05 mm³/mm² p = 10% Peak material volume

Vmc 0.000266 mm³/mm² p = 10%, q = 80% Core material volume

Vvc 0.000309 mm³/mm² p = 10%, q = 80% Core void volume

Vvv 5.9e-05 mm³/mm² p = 80% Pit void volume

Feature Parameters

Spd 3503 1/mm² pruning = 5% Density of peaks

Spc 165 1/mm pruning = 5% Arithmetic mean peak curvature

S10z 3.52 µm pruning = 5% Ten point height

S5p 1.68 µm pruning = 5% Five point peak height

S5v 1.84 µm pruning = 5% Five point pit height

Sda 0.000295 mm² pruning = 5% Mean dale area

Sha 0.000278 mm² pruning = 5% Mean hill area

Sdv 1.27e-08 mm³ pruning = 5% Mean dale volume

Shv 9.13e-09 mm³ pruning = 5% Mean hill volume

Functional Parameters (Stratified surfaces)

Sk 0.645 µm Core roughness depth

Spk 0.377 µm Reduced summit height

Svk 0.583 µm Reduced valley depth

Smr1 8.57 % Upper bearing area

Smr2 82.8 % Lower bearing area

Spq 0.260 Plateau root-mean-square roughness

Svq 0.728 Valley root-mean-square roughness

Smq 87.2 Material ratio at plateau-to-valley transition

EUR 15178N

Hybrid Parameters

Sdq 0.156 Root-mean-square slope

Sds 13342 1/mm² density of summits

Ssc 114 1/mm Arithmetic mean summit curvature

Sdr 1.21 % Developed interfacial area

Sfd 2.65 Fractal dimension of the surface

**Supplementary Table 8.** Surface roughness parameters of the **post-test Load** specimen.

ISO 25178

Height Parameters

Sq 0.302 µm Root-mean-square height

Ssk -1.55 Skewness

Sku 7.36 Kurtosis

Sp 1.05 µm Maximum peak height

Sv 2.23 µm Maximum pit height

Sz 3.28 µm Maximum height

Sa 0.220 µm Arithmetic mean height

Functional Parameters

Smr 52.8 % c = 1 µm under the highest peak Areal material ratio

Smc 0.290 µm p = 10% Inverse areal material ratio

Sxp 0.847 µm p = 50%, q = 97.5% Extreme peak height

Spatial Parameters

Sal 0.0112 mm s = 0.2 Autocorrelation length

Str 0.750 s = 0.2 Texture-aspect ratio

Std 84.3 ° Reference angle = 0° Texture direction

Hybrid Parameters

Sdq 0.135 Root-mean-square gradient

Sdr 0.900 % Developed interfacial area ratio

Functional Parameters (Volume)

Vm 9.89e-06 mm³/mm² p = 10% Material volume

Vv 0.000299 mm³/mm² p = 10% Void volume

Vmp 9.89e-06 mm³/mm² p = 10% Peak material volume

Vmc 0.000233 mm³/mm² p = 10%, q = 80% Core material volume

Vvc 0.000243 mm³/mm² p = 10%, q = 80% Core void volume

Vvv 5.63e-05 mm³/mm² p = 80% Pit void volume

Feature Parameters

Spd 3945 1/mm² pruning = 5% Density of peaks

Spc 144 1/mm pruning = 5% Arithmetic mean peak curvature

S10z 2.69 µm pruning = 5% Ten point height

S5p 0.933 µm pruning = 5% Five point peak height

S5v 1.76 µm pruning = 5% Five point pit height

Sda 0.000206 mm² pruning = 5% Mean dale area

Sha 0.000247 mm² pruning = 5% Mean hill area

Sdv 7.65e-09 mm³ pruning = 5% Mean dale volume

Shv 5.05e-09 mm³ pruning = 5% Mean hill volume

Functional Parameters (Stratified surfaces)

Sk 0.501 µm Core roughness depth

Spk 0.205 µm Reduced summit height

Svk 0.558 µm Reduced valley depth

Smr1 7.37 % Upper bearing area

Smr2 79.6 % Lower bearing area

Spq 0.224 Plateau root-mean-square roughness

Svq 0.739 Valley root-mean-square roughness

Smq 89.2 Material ratio at plateau-to-valley transition

EUR 15178N

Hybrid Parameters

Sdq 0.135 Root-mean-square slope

Sds 12742 1/mm² density of summits

Ssc 94.0 1/mm Arithmetic mean summit curvature

Sdr 0.908 % Developed interfacial area

Sfd 2.70 Fractal dimension of the surface
